# Supplementary material for: Allogeneic CD33-directed CAR-NKT cells for the treatment of bone marrow-resident myeloid malignancies
Source: Nat Commun. 2025 Feb 1;16:1248. doi: 10.1038/s41467-025-56270-6 (PMC11787387; doi:10.1038/s41467-025-56270-6)
Supplement: Supplementary file 1 — Supplementary Information [file 41467_2025_56270_MOESM1_ESM.pdf]

Supplementary Information

Allogeneic CD33-directed CAR-NKT cells for the treatment of bone marrow-resident myeloid malignancies

Yan-Ruide Li[1,2,*], Ying Fang[1,2,*], Siyue Niu[1,2,*], Yichen Zhu[1,2,*], Yuning Chen[1,2], Zibai Lyu[1,2], Enbo Zhu[1,3], Yanxin Tian[1,2], Jie Huang[1,2], Valerie Rezek[4,5,6], Scott Kitchen[4,5,6], Tzung Hsiai[3], Jin J. Zhou[7], Pin Wang[8], Wanxing Chai-Ho[4], Sunmin Park[4], Christopher S. Seet[4,5,9,10], Caspian Oliai[4], Lili Yang[1,2,5,9,10,11,§]

Author Affiliation:

[1]Department of Microbiology, Immunology & Molecular Genetics, University of California, Los Angeles, Los Angeles, CA 90095, USA

[2]Department of Bioengineering, University of California, Los Angeles, Los Angeles, CA 90095, USA

[3]Division of Cardiology, Department of Medicine, David Geffen School of Medicine, University of California, Los Angeles, Los Angeles, CA 90095, USA

[4]Division of Hematology-Oncology, Department of Medicine, David Geffen School of Medicine, University of California, Los Angeles, Los Angeles, CA 90095, USA

[5]The Eli and Edythe Broad Center of Regenerative Medicine and Stem Cell Research, University of California, Los Angeles, Los Angeles, CA 90095, USA

[6]UCLA AIDS Institute, David Geffen School of Medicine, University of California, Los Angeles, Los Angeles, CA 90095, USA

[7]Department of Biostatistics, Fielding School of Public Health, University of California, Los Angeles, Los Angeles, CA 90095, USA

[8]Department of Chemical Engineering and Materials Science, University of Southern California, Los Angeles, CA 90089, USA

[9]Jonsson Comprehensive Cancer Centre, University of California, Los Angeles, Los Angeles, CA 90095, USA

[10]Molecular Biology Institute, University of California, Los Angeles, Los Angeles, CA 90095, USA

[11]Parker Institute for Cancer Immunotherapy, University of California, Los Angeles, Los Angeles, CA 90095, USA

[*]These authors contributed equally

[§]Corresponding author. Email: liliyang@ucla.edu.

Table of Contents

Supplementary Fig. 1. FACS gating strategies and the transcriptome profiling of primary MDS patient samples, related to Fig. 1. 3
Supplementary Fig. 2. Diagram showing the generation of ^{Allo15}CAR33-NKT cells in the *Ex Vivo* HSPC-Derived CAR-NKT Cell Culture. 4
Supplementary Fig. 3. Generation of HSPC-engineered ^{Allo15}CAR33-NKT cells, related to Fig. 2. 5
Supplementary Fig. 4. Generation of ^{Allo15}CAR33-NKT cells using 2-vector system, related to Fig. 2. 6
Supplementary Fig. 5. Generation of CAR33-T cells and comparison between ^{Allo15}CAR33-NKT and CAR33-T cells, related to Fig. 2. 8
Supplementary Fig. 6. Comparison of ^{Allo15}CAR33-NKT cells generated using three expansion approaches, related to Fig. 2. 9
Supplementary Fig. 7. *In vitro* antitumor efficacy and mechanism of action (MOA) study of ^{Allo15}CAR33-NKT cells, related to Fig. 3. 10
Supplementary Fig. 8. *In vivo* antitumor efficacy of ^{Allo15}CAR33-NKT cells using human AML xenograft mouse models, related to Fig. 5. 11
Supplementary Fig. 9. Studying the phenotype and functionality of ^{Allo15}CAR33-NKT cells in a THP1-FG human AML xenograft NSG mouse model, related to Fig. 5a-5g. 12
Supplementary Fig. 10. Gene profiles of ^{Allo15}CAR33-NKT cells after repeated challenges with AML tumor cells. 13
Supplementary Fig.11. Gene profiling of ^{Allo15}CAR33-NKT and CAR33-T cells. 15
Supplementary Fig. 12. Studying the *in vivo* synergistic effect of ^{Allo15}CAR33-NKT cells with HMA in a KG1-FG human AML xenograft NSG mouse model, related to Fig. 7. 16
Supplementary Fig. 13. CD33 expression on various human tissue cells and characterization of BLT mice, related to Fig. 8. 17
Supplementary Fig. 14. Study the immunogenicity of ^{Allo15}CAR33-NKT cells. 19
Supplementary Fig. 15. Safety study of ^{15}CAR33-T cells, related to Fig. 10. 21
Supplementary Table 1. Information on clinical primary AML and MDS patient samples. 22
Supplementary Table 2. Cancer stem cell (CSC) and natural killer receptor (NKR) ligand gene signatures 23

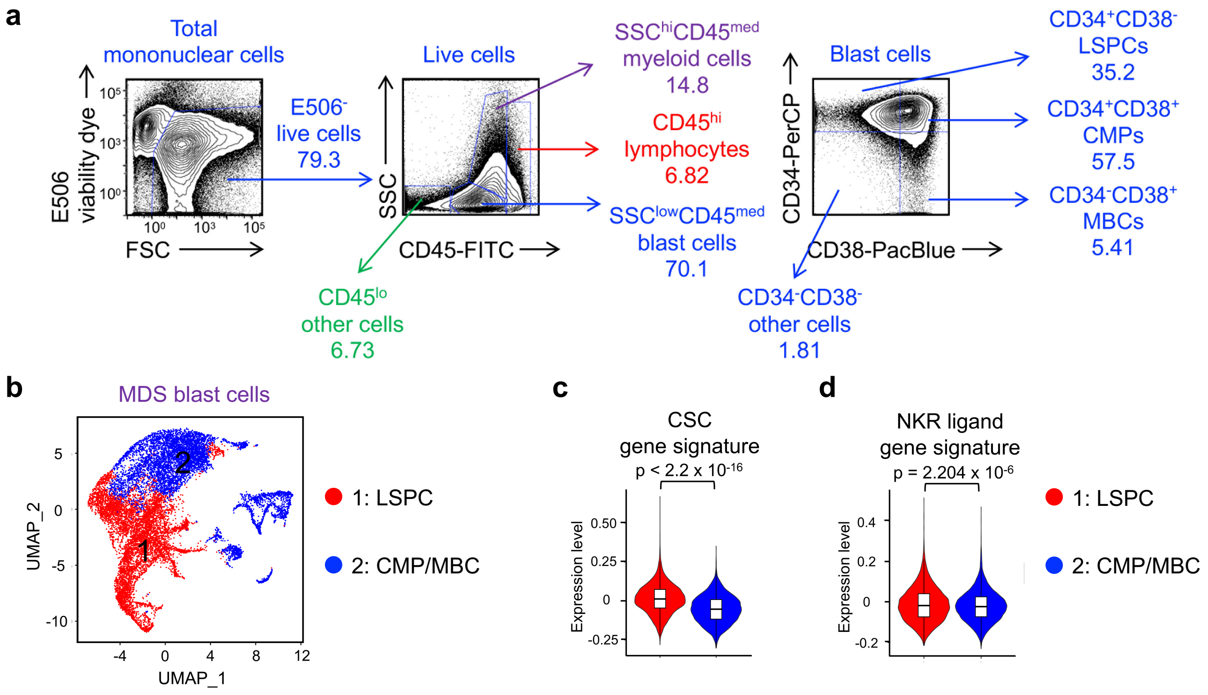

Supplementary Fig. 1. FACS gating strategies and the transcriptome profiling of primary MDS patient samples, related to Fig. 1.

a. FACS gating strategies for analyzing blast cells and their subpopulations in primary patient bone marrow samples. One representative data from AML samples #4 are presented. The gating panels correspond to main **Fig. 1c-e**.

b-d. Transcriptome profiling of primary MDS patient samples. Data from NCBI Sequence Read Archive (PRJNA720840) were included for the scRNA-seq analyses. 5 primary MDS blast samples were analyzed.

b. Combined UMAP plot showing the formation of two major cell clusters.

c and **d**. Expression of CSC gene signature (**c**) and NKR ligand gene signature (**d**) in the indicated cell clusters. Violin plots showing the gene expression levels are presented. Box and whisker plots exhibit the minimum, lower quartile, median, upper quartile and maximum expression levels of each type of cell. p values of violin plots were determined by Wilcoxon rank sum test.

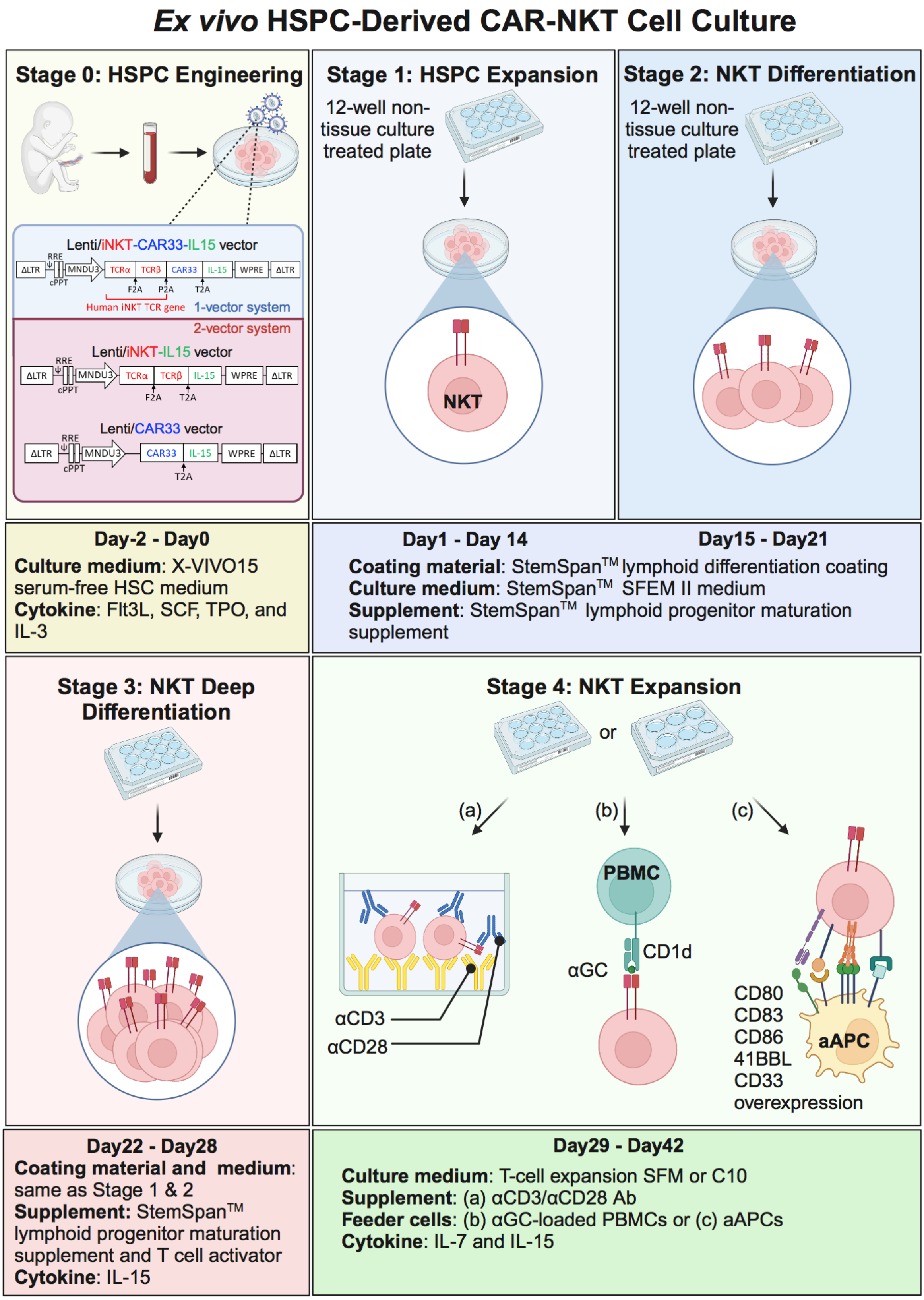

Supplementary Fig. 2. Diagram showing the generation of ^{Allo15}CAR33-NKT cells in the *Ex Vivo* HSPC-Derived CAR-NKT Cell Culture.
Created in BioRender. FANG, Y. (2025) https://BioRender.com/a10e121

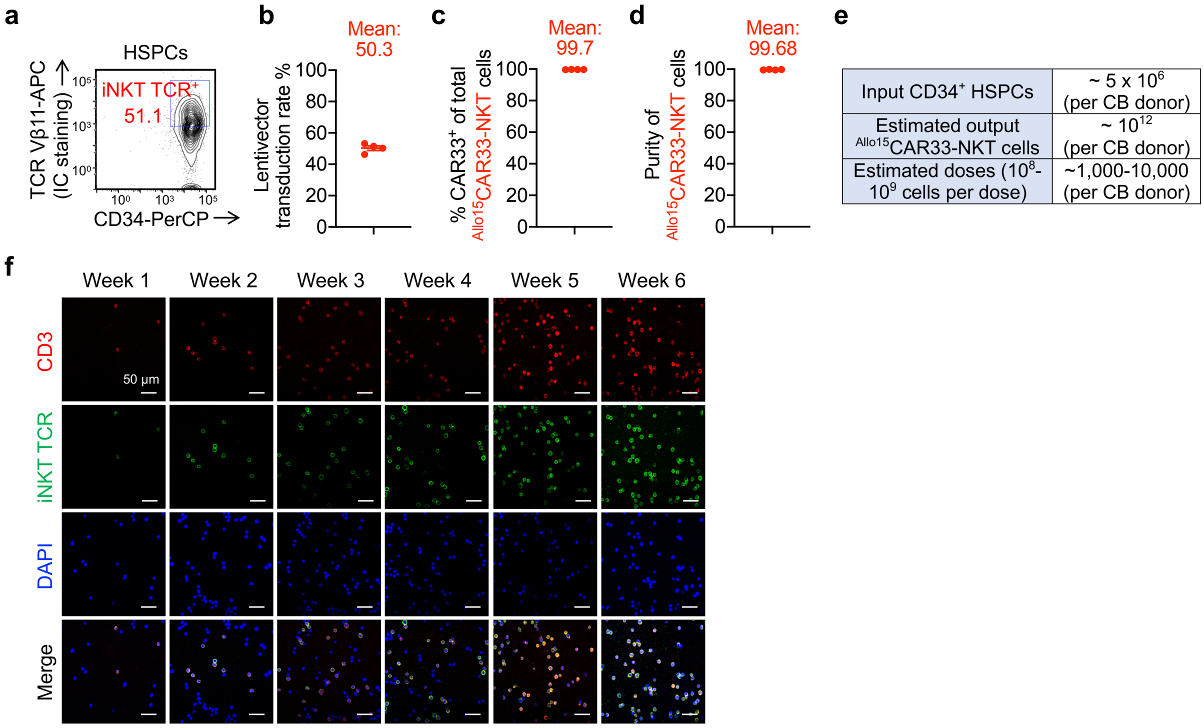

Input CD34+ HSPCs	~ 5 x 10^6 (per CB donor)
Estimated output Allo15CAR33-NKT cells	~ 10^{12} (per CB donor)
Estimated doses (10^8-10^9 cells per dose)	~1,000-10,000 (per CB donor)

Supplementary Fig. 3. Generation of HSPC-engineered ^{Allo15}CAR33-NKT cells, related to Fig. 2.

a. Intracellular expression of iNKT TCR (identified as Vβ11$^+$) in the engineered cord blood HSPCs 72 hours after lentivector transduction.

b. Quantification of **a** (n = 4; n indicates different cord blood donors).

c. CD33-targeting CAR expression on ^{Allo15}CAR33-NKT cells (n = 4; n indicates different cord blood donors).

d. Purity of ^{Allo15}CAR33-NKT cells (n = 4; n indicates different cord blood donors). The purity of the cells was assessed based on the presence of iNKT TCR$^+$CD3$^+$ markers.

e. Table showing the estimated output cell numbers and doses of ^{Allo15}CAR33-NKT cells.

f. Immunofluorescence monitoring of the generation of ^{Allo15}CAR33-NKT cells during the 6-week culture. iNKT TCR was stained using a 6B11 monoclonal antibody. Note that the CD3 and DAPI panels were also presented in the main **Fig. 2c**.

Representative of > 5 experiments. Data are presented as the mean ± SEM. Source data and exact *p* values are provided as a Source Data file.

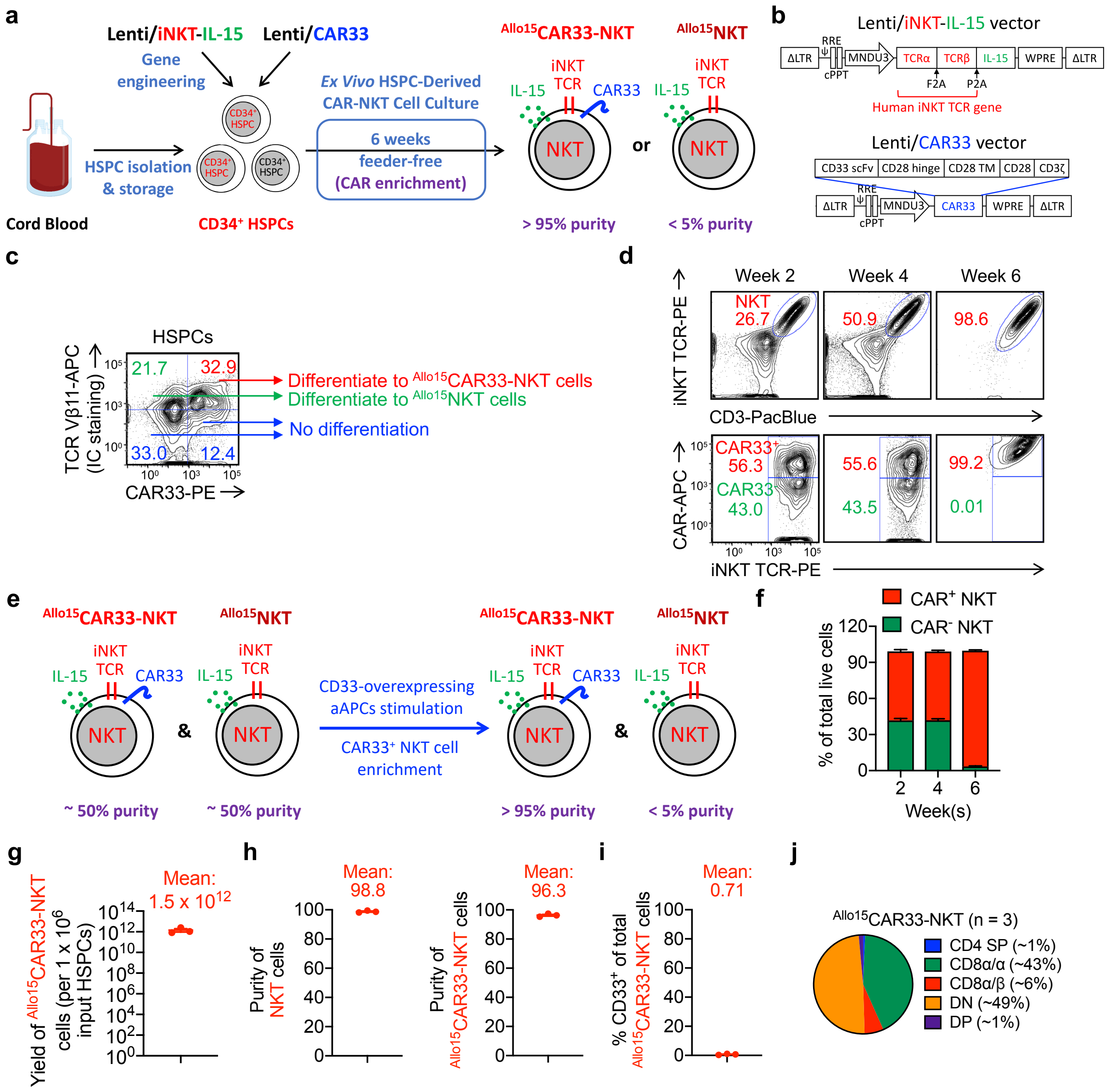

Supplementary Fig. 4. Generation of ^{Allo15}CAR33-NKT cells using 2-vector system, related to Fig. 2.

a. Schematics showing the generation of ^{Allo15}CAR33-NKT cells using the 2-vector system. Created in BioRender. LI, Y. (2025) https://BioRender.com/n50o257

b. Schematics showing the design of Lenti/iNKT-IL-15 and Lenti/CAR33 lentivectors.

c. Intracellular expression of iNKT TCR (identified as Vβ11^{+}) and surface expression of CAR33 in CB HSPCs 72 h after lentivector transduction. Note that HSPCs that are successfully transduced with both lentivectors develop into ^{Allo15}CAR33-NKT cells, those transduced solely with Lenti/iNKT-IL-15 lentivector differentiate into ^{Allo15}NKT cells, while those transduced with Lenti/CAR33 or not transduced do not differentiate into NKT cells.

d. FACS monitoring of the generation of ^{Allo15}CAR33-NKT and ^{Allo15}NKT cells. iNKT TCR was stained using a 6B11 monoclonal antibody.

e. Schematics showing the enrichment of ^{Allo15}CAR33-NKT cells using CD33-overexpressing aAPCs.

f. The percentage of ^{Allo15}CAR33-NKT and ^{Allo15}NKT cells during the 6-week culture (n = 3; n indicates different cell batches).

g. Yield of ^{Allo15}CAR33-NKT cells generated using 2-vector system (n = 3; n indicates different cell batches).

h. Purity of total NKT and $^{\text{Allo15}}$CAR33-NKT cells (n = 3; n indicates different cell batches). The purity of NKT cells was assessed based on the presence of iNKT TCR^{+}CD3^{+} markers, and the purity of $^{\text{Allo15}}$CAR33-NKT cells was assessed based on the presence of iNKT TCR^{+}CD3^{+}CAR33^{+} markers.
i. CD33 expression on $^{\text{Allo15}}$CAR33-NKT cells (n = 3; n indicates different cell batches).
j. CD4/CD8 subpopulation percentages of $^{\text{Allo15}}$CAR33-NKT cells (n = 3; n indicates different cell batches)
Representative of 3 experiments. Data are presented as the mean ± SEM. Source data and exact *p* values are provided as a Source Data file.

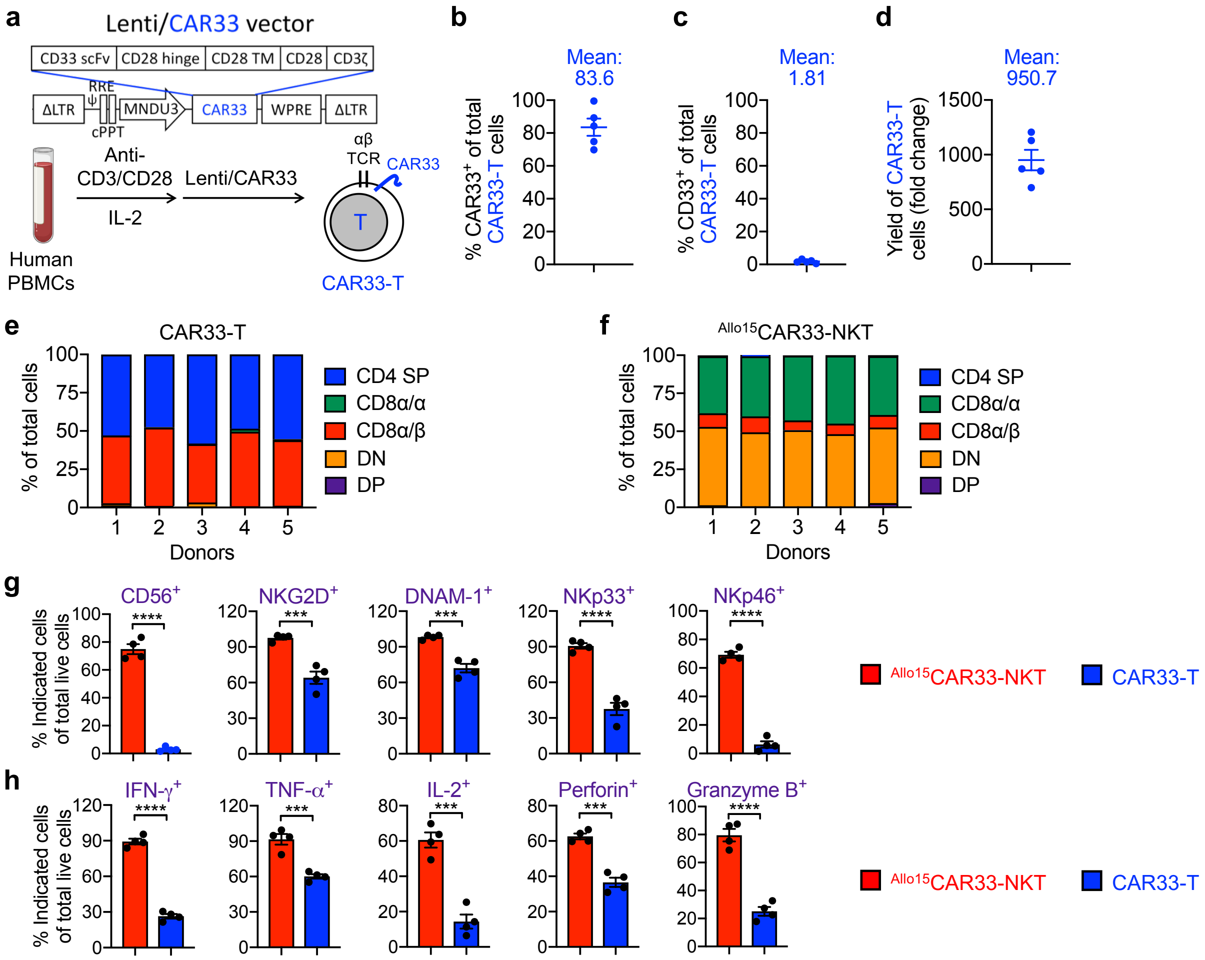

Supplementary Fig. 5. Generation of CAR33-T cells and comparison between ^{Allo15}CAR33-NKT and CAR33-T cells, related to Fig. 2.

a. Diagram showing the design of Lenti/CAR33 lentivector, and the generation of CD33-taregting CAR-T (CAR33-T) cells from healthy donor peripheral blood mononuclear cells (PBMCs). Created in BioRender. LI, Y. (2025) https://BioRender.com/o37h997

b. CD33-targeting CAR expression on CAR33-T cells (n = 5; n indicates different cell batches).

c. CD33 expression on CAR33-T cells (n = 5; n indicates different cell batches).

d. Yield of CAR33-T cells (n = 5; n indicates different PBMC donors).

e. Comparison of CD4/CD8 subpopulation percentages of CAR33-T cells. Data from 5 different cell batches were shown.

f. Comparison of CD4/CD8 subpopulation percentages of ^{Allo15}CAR33-NKT cells. Data from 5 different cell batches were shown.

g. FACS quantification of NK marker and NKR expressions on ^{Allo15}CAR33-NKT and CAR33-T cells (n = 4; n indicates different cell batches).

h. FACS quantification of intracellular cytokine and cytotoxic molecule production by ^{Allo15}CAR33-NKT and CAR33-T cells (n = 4; n indicates different cell batches).

Representative of > 5 experiments. Data are presented as the mean ± SEM. ***p < 0.001; ****p < 0.0001, by Student's *t* test (**g** and **h**). Source data and exact *p* values are provided as a Source Data file.

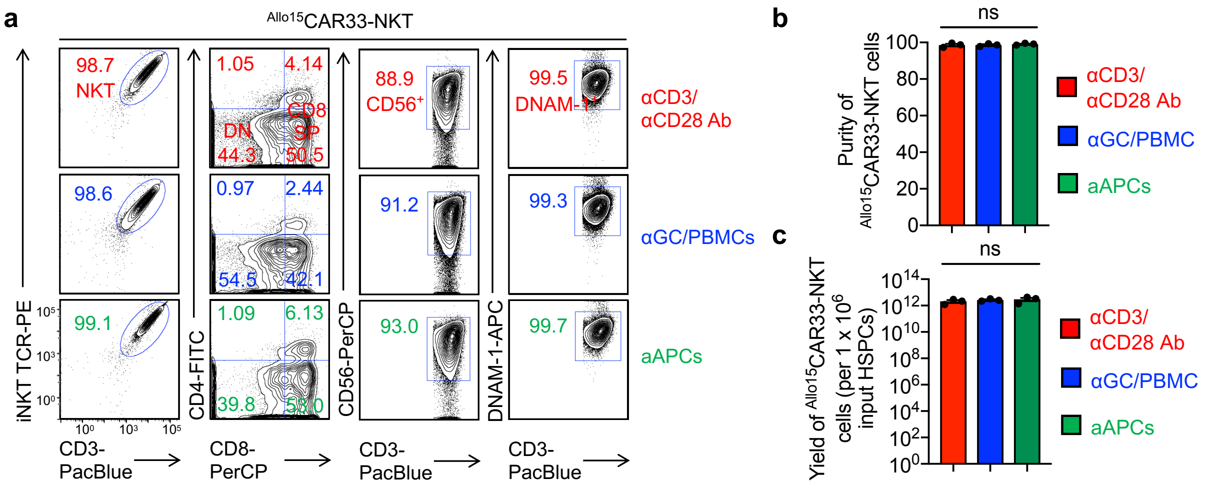

Supplementary Fig. 6. Comparison of ^{Allo15}CAR33-NKT cells generated using three expansion approaches, related to Fig. 2.

a. FACS detection of surface markers on ^{Allo15}CAR33-NKT cells generated using three expansion approaches. The three expansion approaches are αCD3/αcCD28 Ab, αGC/PBMCs, and aAPCs.

b. Comparison of ^{Allo15}CAR33-NKT cell purity generated using the three expansion approaches (n = 3; n indicates different cell batches). The purity of the cells was assessed based on the presence of iNKT TCR^{+}CD3^{+} markers.

c. Comparison of ^{Allo15}CAR33-NKT cell yield generated using the three expansion approaches (n = 3; n indicates different cell batches).

Representative of 3 experiments. Data are presented as the mean ± SEM. ns, not significant by one-way ANOVA (**b** and **c**). Source data and exact *p* values are provided as a Source Data file.

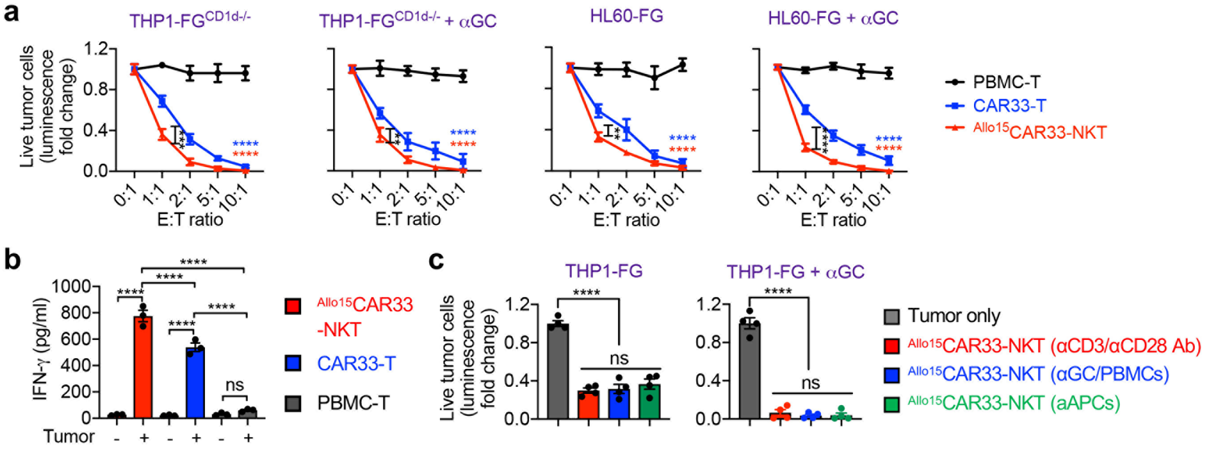

Supplementary Fig. 7. *In vitro* antitumor efficacy and mechanism of action (MOA) study of ^{Allo15}CAR33-NKT cells, related to Fig. 3.

a. Studying the antitumor efficacy of ^{Allo15}CAR33-NKT cells against multiple AML tumor cell lines. Tumor cell killing data at 24 h (n = 4).

b. ELISA analyses of IFN-γ production by the indicated therapeutic cells (n = 4).

c. Comparison of the *in vitro* antitumor efficacy of ^{Allo15}CAR33-NKT cells generated using the three expansion approaches (n = 4).

Representative of 3 experiments. Data are presented as the mean ± SEM. ns, not significant; **p < 0.01; ***p < 0.001; ****p < 0.0001 by one-way ANOVA (**b** and **c**), or two-way ANOVA (**a**). Source data and exact *p* values are provided as a Source Data file.

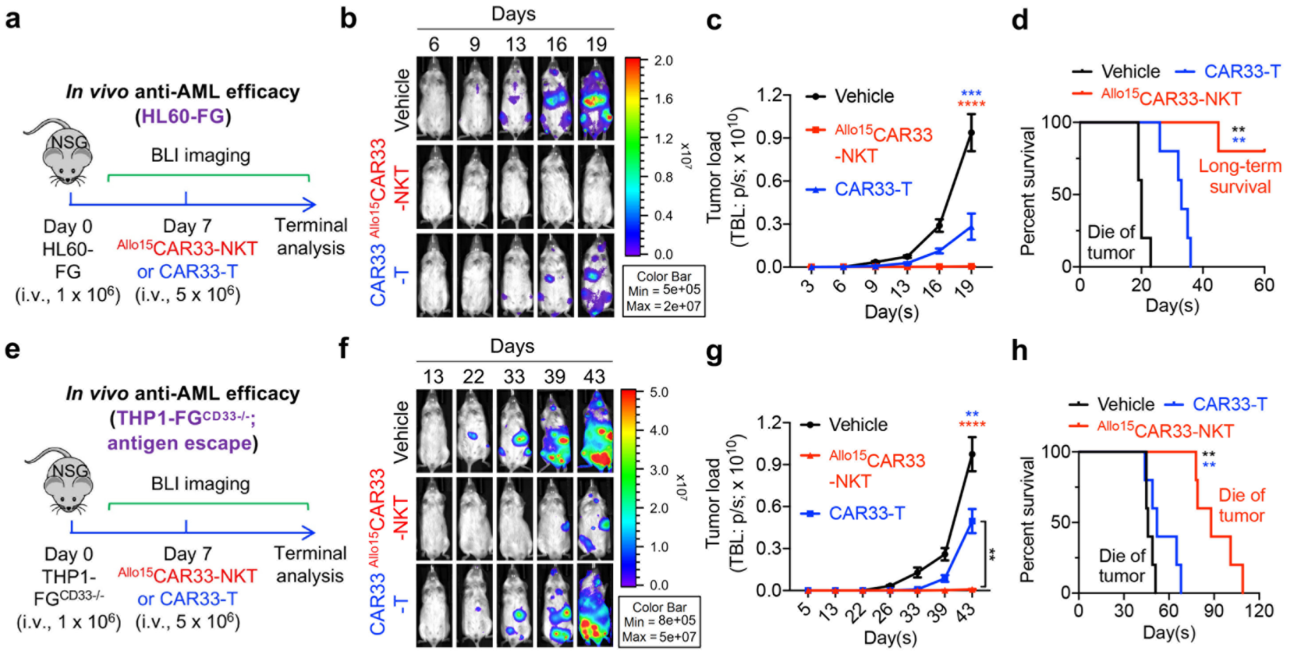

Supplementary Fig. 8. ***In vivo* antitumor efficacy of ^{Allo15}CAR33-NKT cells using human AML xenograft mouse models, related to Fig. 5.**

a-**d**. Studying the *in vivo* antitumor efficacy of ^{Allo15}CAR33-NKT cells using an HL60-FG human AML xenograft NSG mouse model. **a**. Experimental design. **b**. BLI images showing the presence of tumor cells in experimental mice over time. **c**. Quantification of **b** (n = 5). TBL, total body luminescence. **d**. Kaplan-Meier survival curves of experimental mice over time (n = 5).

e-**h**. Studying the *in vivo* antitumor efficacy of ^{Allo15}CAR33-NKT cells using a THP1-FG$^{CD33-/-}$ human AML xenograft NSG mouse model. The THP1-FG$^{CD33-/-}$ tumor cell line is used to mimic CAR antigen escape in AML tumor cells. **e**. Experimental design. **f**. BLI images showing the presence of tumor cells in experimental mice over time. **g**. Quantification of **f** (n = 5). **h**. Kaplan-Meier survival curves of experimental mice over time (n = 5).

Representative of 2 experiments. Data are presented as the mean ± SEM. ns, not significant; *p < 0.05; **p < 0.01; ***p < 0.001; ****p < 0.0001, by one-way ANOVA (**c** and **g**) or log rank (Mantel-Cox) text adjusted for multiple comparisons (**d** and **h**). Source data and exact *p* values are provided as a Source Data file.

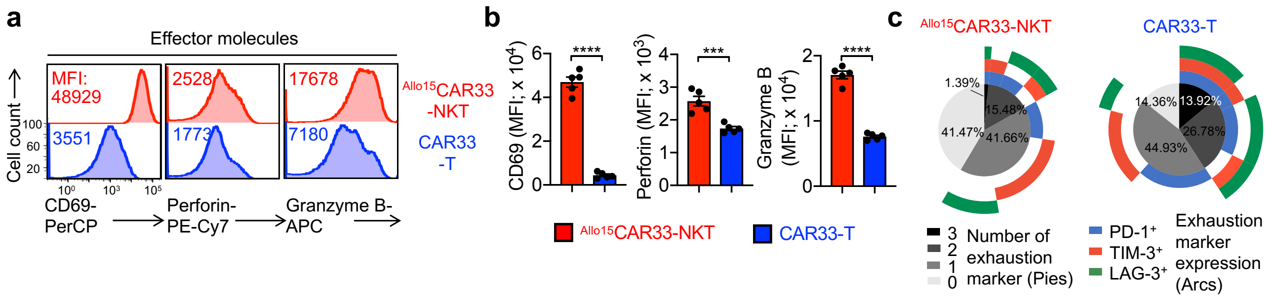

Supplementary Fig. 9. Studying the phenotype and functionality of ^{Allo15}CAR33-NKT cells in a THP1-FG human AML xenograft NSG mouse model, related to Fig. 5a-5g.

a. FACS detection of surface CD69 as well as intracellular Perforin and Granzyme B in therapeutic cells isolated from mouse liver collected on day 20.

b, Quantification of **a** (n = 5).

c, SPICE analysis of exhaustion marker expression of the indicated therapeutic cells. Pie charts reflect proportions of indicated cell groups expressing indicated numbers (0-3) of exhaustion markers. Colored arcs indicate the specific combinations of exhaustion markers expressed.

Representative of 2 experiments. Data are presented as the mean ± SEM. ***p < 0.001; ****p < 0.0001, by Student's *t* test (**b**). Source data and exact *p* values are provided as a Source Data file.

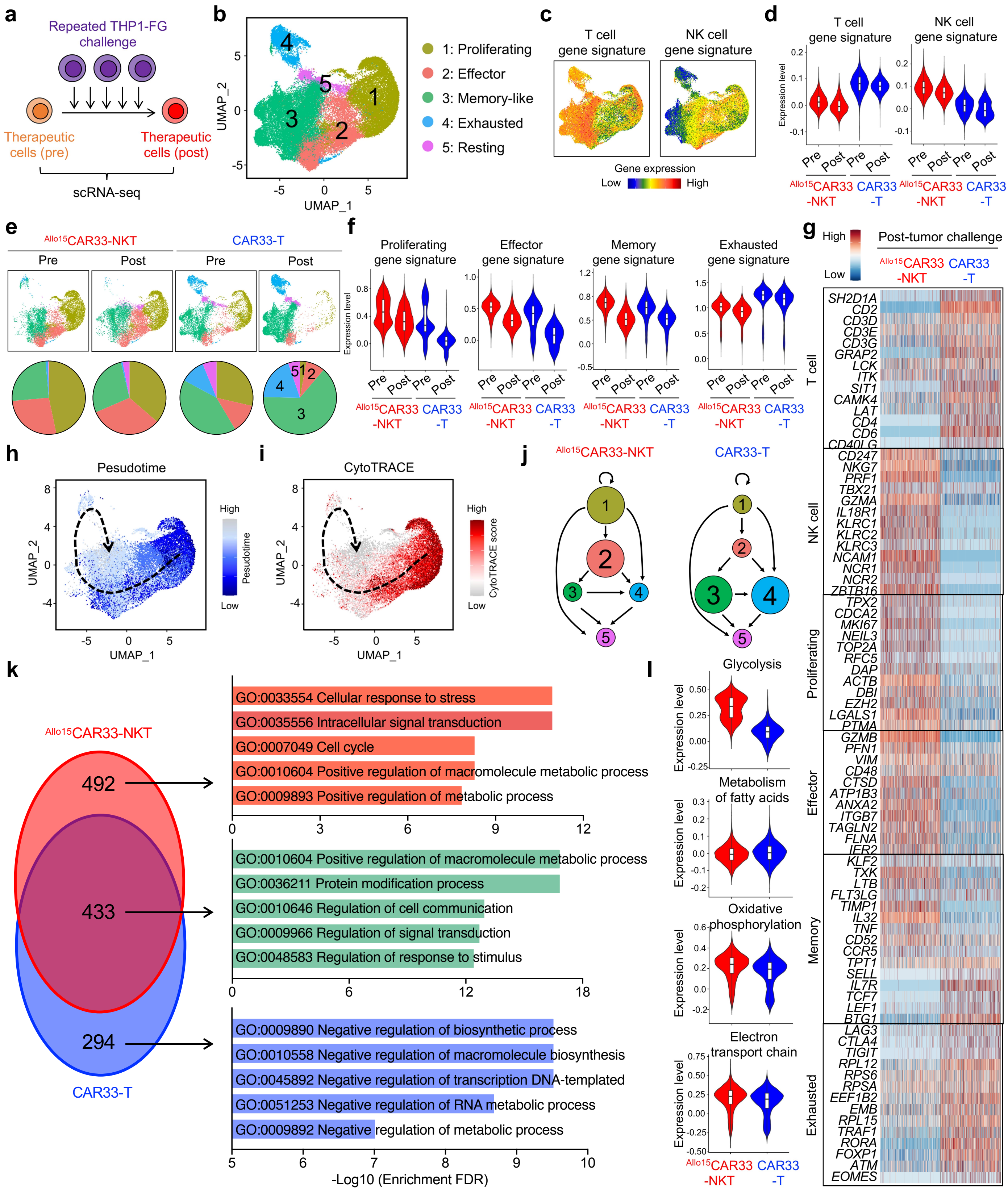

Supplementary Fig. 10. Gene profiles of ^{Allo15}CAR33-NKT cells after repeated challenges with AML tumor cells.

a, Schematic showing the experimental design to study the gene profiling of ^{Allo15}CAR33-NKT cells using scRNA-seq. Conventional CAR33-T cells were included as a control. Therapeutic cells were analyzed before and after the THP1-FG tumor cell challenge.

b, Combined UMAP plot showing the formation of five major cell clusters: Proliferating, Effector, Memory-like, Exhausted, and Resting. Total cells combined from all therapeutic cell samples are included. Each dot represents a single cell and is colored according to its cell cluster assignment.
c, UMAP plots showing the expression distribution of T and NK cell gene signatures in the combined samples.
d, Violin plots showing the expression distribution of T and NK cell gene signatures in the indicated individual samples.
e, Individual UMAP plots showing cell cluster composition of the indicated therapeutic cell samples, and pie charts showing the cell cluster proportions of the indicated therapeutic cell samples.
f, Violin plots showing the expression distribution of proliferating, effector, memory, and exhausted gene signatures in the indicated individual samples.
g, Heatmap showing the expression of representative signature genes. Each column indicates an individual cell. Each row indicates an individual gene.
h, Pseudotime directionality projection on the combined UMAP plots of ^{Allo15}CAR33-NKT cell samples. Each dot represents a single cell and is colored according its assigned pseudotime value. Dotted lines indicate the pseudotime trajectories.
i, CytoTRACE scores projection on the combined UMAP plots of ^{Allo15}CAR33-NKT cell samples. Each dot represents a single cell and is colored according its assigned CytoTRACE score. Dotted lines indicate the differentiation trajectories.
j, Diagrams summarizing the UMAP cell clustering, Pseudotime trajectory, and CytoTRACE analyses. The cell cluster proportions are represented by the size of circles, and the transitions between cell clusters are symbolized using connecting arrows.
k, Venn diagram illustrating the numbers of shared and unique pathways of ^{Allo15}CAR33-NKT and CAR33-T cell samples. The pathway analyses were conducted by comparing differentially expressed genes between post-tumor cell challenge samples and pre-tumor cell challenge samples. The indicated pathway in each category are shown in the bar plots.
l, Violin plots showing the expression levels of the indicated metabolic pathways in ^{Allo15}CAR33-NKT cell and CAR33-T cell samples.
The experiment was performed once; cells collected from three repeated experiments were combined for analyses. In the violin plots (**d, f,** and **l**), box and whisker plots exhibit the minimum, lower quartile, median, upper quartile and maximum expression levels of each type of cell. One ^{Allo15}CAR33-NKT sample before tumor cell challenge (containing 12,006 cells), one ^{Allo15}CAR33-NKT sample after tumor cell challenge (containing 10,418 cells), one CAR33-T sample before tumor cell challenge (containing 9,122 cells), and one CAR33-T sample after tumor cell challenge (containing 9,949 cells) were analyzed. Source data and exact *p* values are provided as a Source Data file.

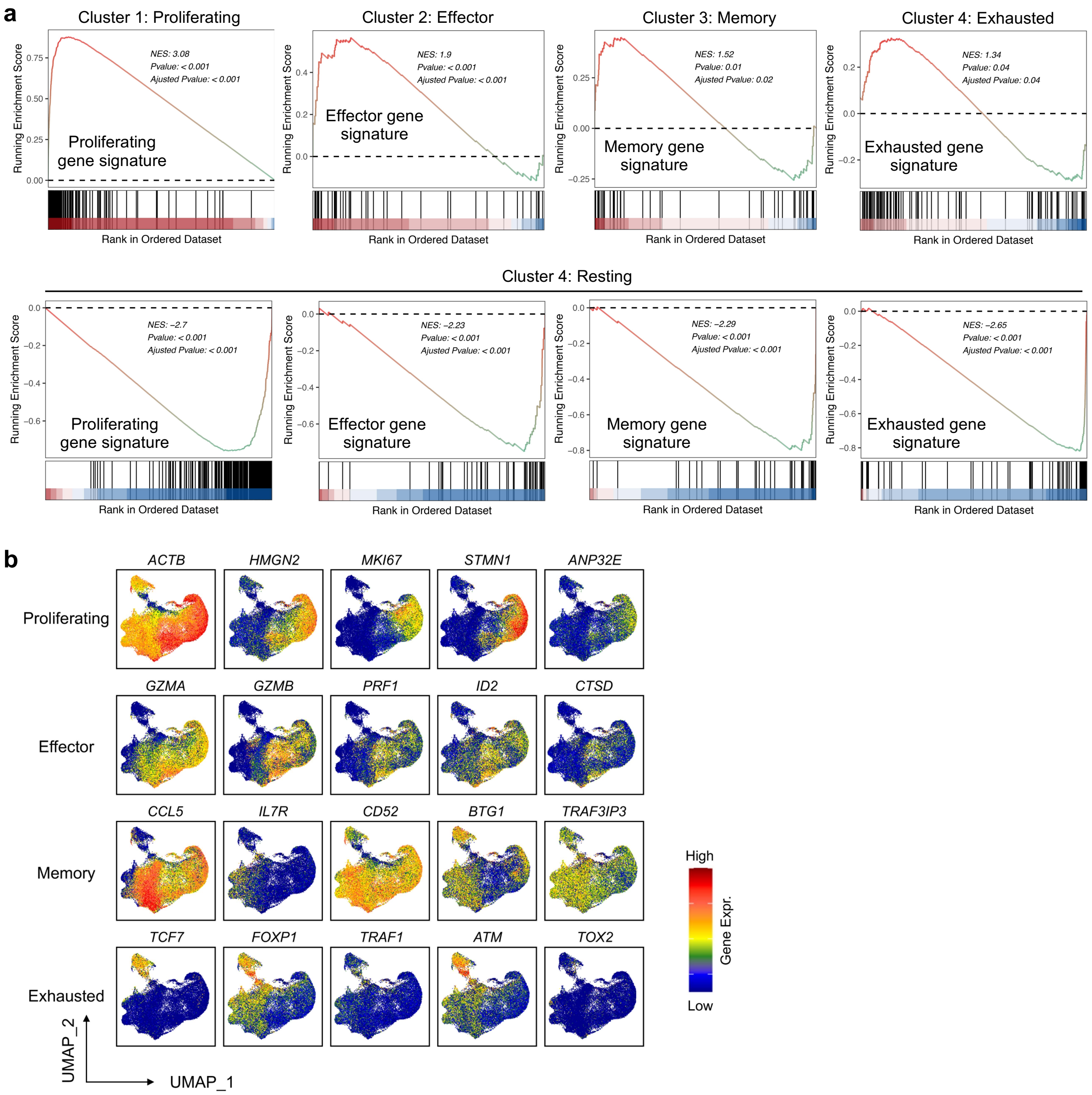

Supplementary Fig.11. Gene profiling of ^{Allo15}CAR33-NKT and CAR33-T cells.
a. Gene Set Enrichment Analysis (GSEA) plots showing the enrichment of gene signatures of proliferating, effector, memory, and exhausted cells in the indicated cell clusters. Normal p value calculated as two-tailed *t*-test.
b. UMAP plots showing the expression of the indicated signature genes.
The experiment was performed once; cells collected from three repeated experiments were combined for analyses. One ^{Allo15}CAR33-NKT sample before tumor cell challenge (containing 12,006 cells), one ^{Allo15}CAR33-NKT sample after tumor cell challenge (containing 10,418 cells), one CAR33-T sample before tumor cell challenge (containing 9,122 cells), and one CAR33-T sample after tumor cell challenge (containing 9,949 cells) were analyzed.

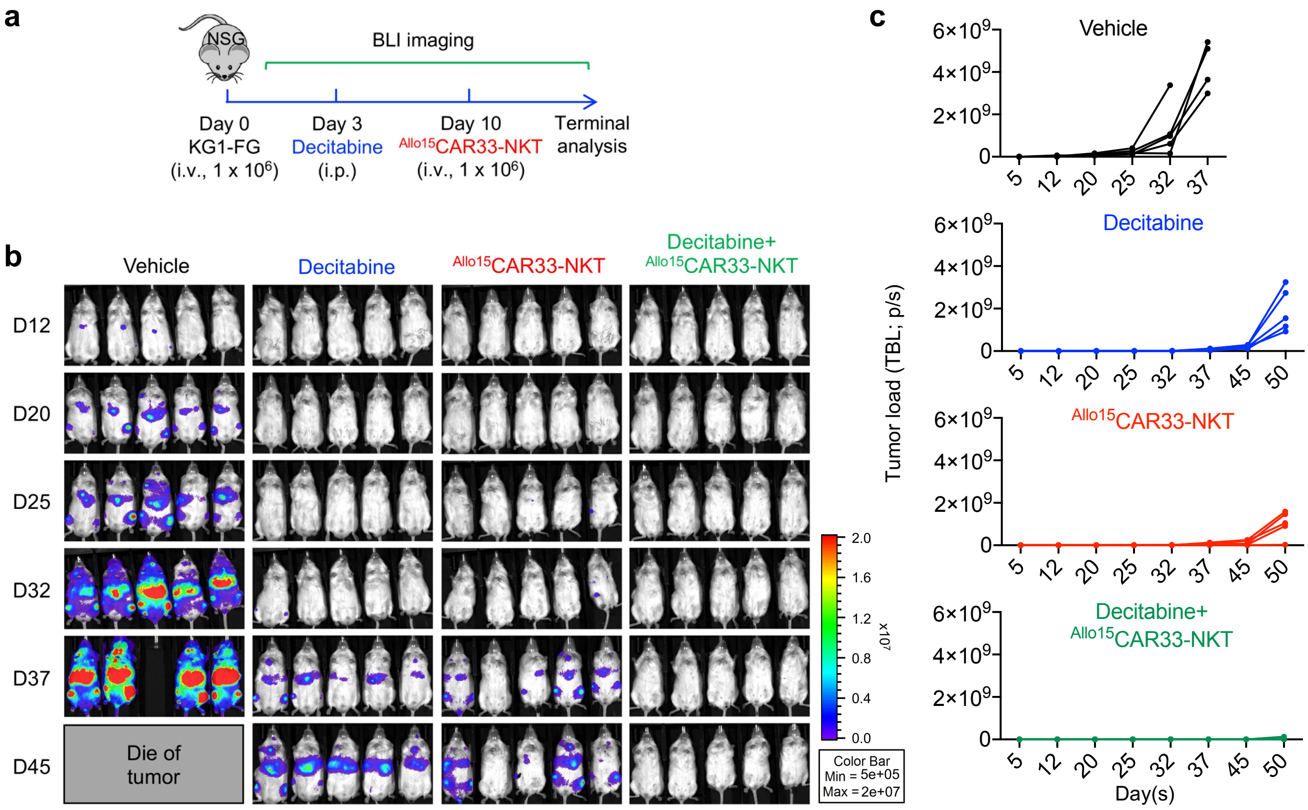

Supplementary Fig. 12. Studying the *in vivo* synergistic effect of ^{Allo15}CAR33-NKT cells with HMA in a KG1-FG human AML xenograft NSG mouse model, related to Fig. 7.
a. Experimental design.
b. BLI images showing the presence of tumor cells in experimental mice over time.
c. Quantification of **b** (n = 5).
Representative of 2 experiments. Source data and exact *p* values are provided as a Source Data file.

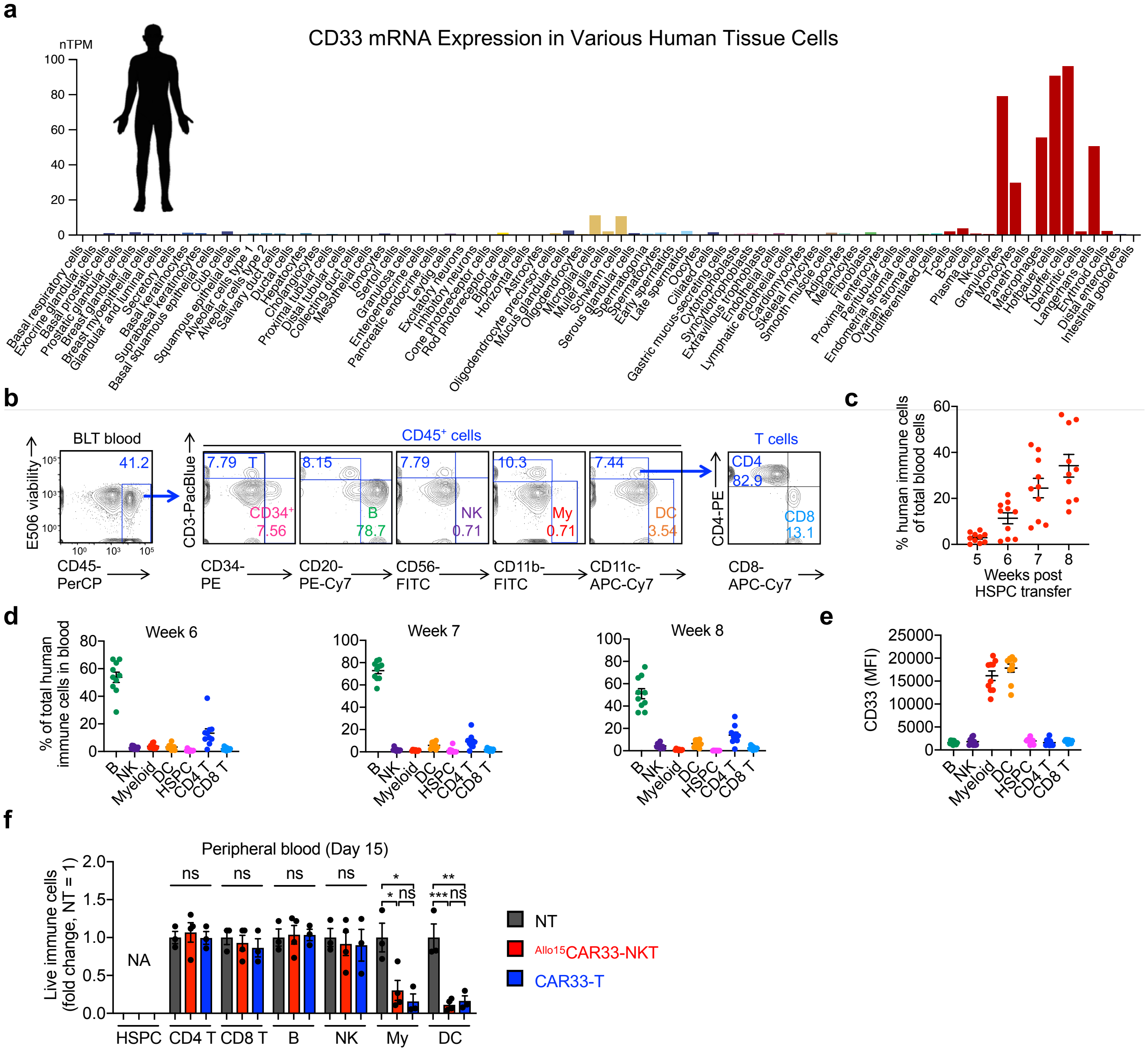

Supplementary Fig. 13. CD33 expression on various human tissue cells and characterization of BLT mice, related to Fig. 8.

a. CD33 mRNA expression in various human tissue cells. Data were adapted from the Human Protein Atlas (https://www.proteinatlas.org/ENSG00000105383-CD33). nTPM, normalized transcripts per million. Created in BioRender. LI, Y. (2025) https://BioRender.com/n50o257

b-d. Characterization of BLT mice. **b**. FACS detection of human immune cells in the peripheral blood collected from BLT mice 8 weeks post HSPC injection and prior to therapeutic cell (i.e., ^{Allo15}CAR33-NKT and CAR33-T cells) injection. My, myeloid cell; DC, dendritic cell. **c**. FACS quantification of the percentage of human immune cells out of total blood cells at the indicated weeks (n = 10). Human immune cells were identified as human CD45^{+} cells. **d**. FACS quantification of the percentage of the indicated human immune cells out of total human CD45^{+} cells (n = 10).

e. FACS analyses of CD33 expression on the indicated immune cells in the peripheral blood collected from BLT mice 8 weeks post HSPC injection and prior to therapeutic cell injection (n = 10).

f. FACS analyses of immune cell targeting by therapeutic cells in the peripheral blood 15 days post-injection of therapeutic cells (n = 3 for NT and CAR33-T, and n = 4 for ^{Allo15}CAR33-NKT). The percentage of the indicated immune cells among total CD45^{+}CAR^{-} immune cells from

each experimental mouse was recorded, and the fold change was calculated by normalizing to the NT group. NA, not available.

Data are presented as the mean ± SEM. ns, not significant; $*p < 0.05$; $**p < 0.01$; $***p < 0.001$; by one-way ANOVA (**f**). Source data and exact *p* values are provided as a Source Data file.

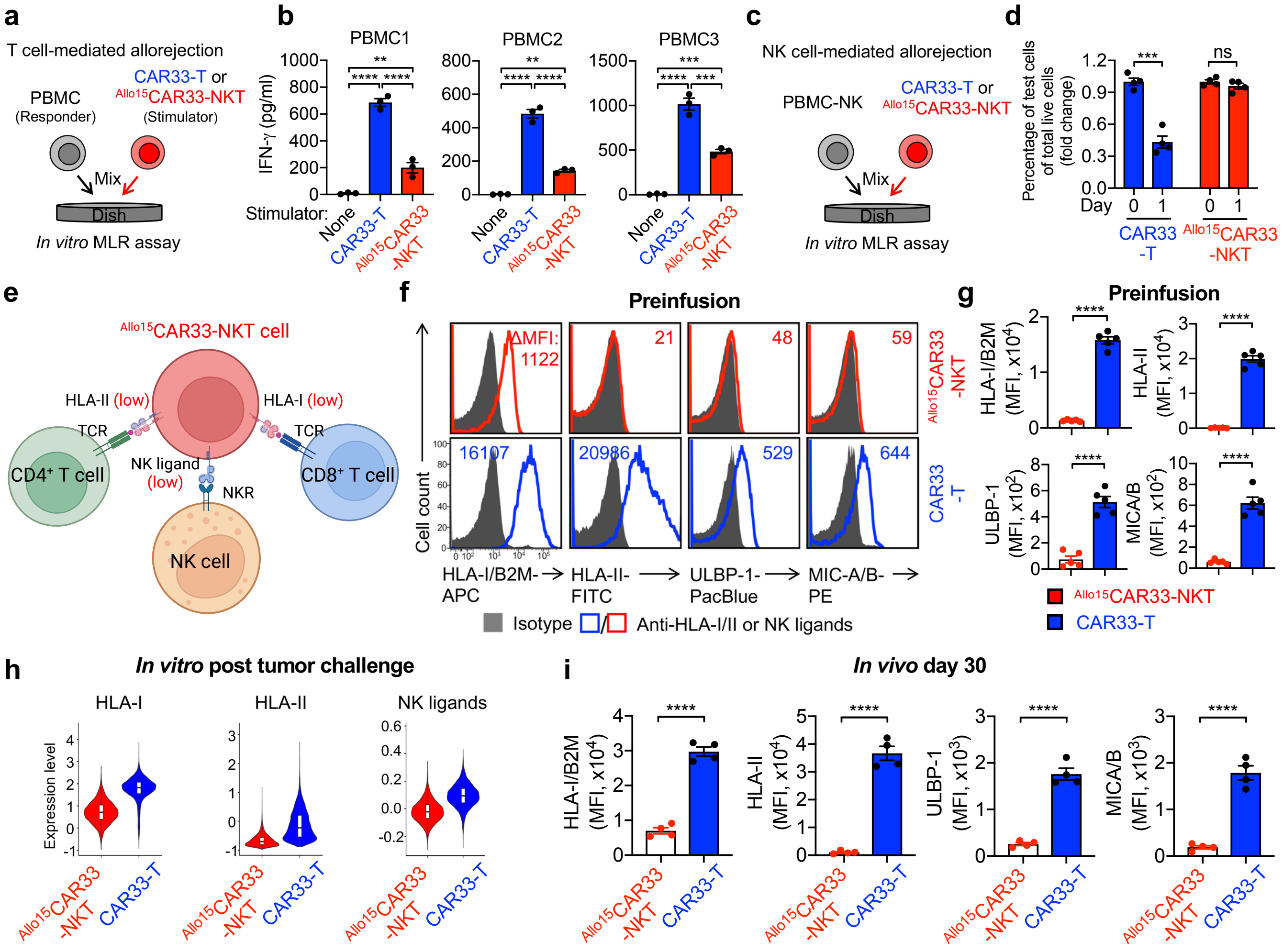

Supplementary Fig. 14. Study the immunogenicity of ^{Allo15}CAR33-NKT cells.
a and **b.** Studying the T cell-mediated allorejection against ^{Allo15}CAR33-NKT cells using an *in vitro* mixed lymphocyte reaction (MLR) assay. PBMCs from over 5 random mismatched healthy donors were used as responder cells, and irradiated ^{Allo15}CAR33-NKT cells were used as stimulator cells. Data from three representative donors are presented. CAR33-T cells were included as an allorejection control. **a**. Experimental design. **b**. ELISA analyses of IFN-γ production on day 4 (n = 3).
c and **d**. Studying the NK cell-mediated allorejection against ^{Allo15}CAR33-NKT cells using an *in vitro* MLR assay. PBMC-NK cells isolated from over 5 random mismatched healthy donors were used. Data from one representative donor are presented. ^{Allo15}CAR33-NKT and CAR33-T cells were included as allorejection controls. **c**. Experimental design. **d**. FACS quantification of the indicated live cells on day 0 and day 1 (n = 3).
e. Illustration depicting the hypoimmunogenecity working model of ^{Allo15}CAR33-NKT cells. Created in BioRender. LI, Y. (2025) https://BioRender.com/k95r142
f and **g**. FACS measurements (**f**) and quantifications (**g**) of surface HLA-I/II and NK ligands (i.e., ULBP-1 and MICA/B) on ^{Allo15}CAR33-NKT cells (n = 5; n indicates different cell product batches).
h. scRNA-seq analysis of the cells collected from the *in vitro* THP1-FG tumor repeat challenge assay as described in **Supplementary Fig. 10a**.
i. FACS measurements of surface HLA-I/II and NK ligands (i.e., ULBP-1 and MICA/B) on ^{Allo15}CAR33-NKT cells post *in vivo* antitumor response as described in **Fig. 5a** (n = 4; n indicates different experimental mice).
Representative of 3 experiments. Data are presented as the mean ± SEM. ns, not significant; **p < 0.01; ***p < 0.001; ****p < 0.0001, by Student's *t* test (**d**, **g** and **i**), or one-way ANOVA

(**b**). In the violin plots (**h**), box and whisker plots exhibit the minimum, lower quartile, median, upper quartile and maximum expression levels of each type of cell. For the scRNA-seq analyses (**h**), one ^{Allo15}CAR33-NKT sample (containing 12,006 cells) and one CAR33-T sample (containing 9,122 cells) were analyzed. Source data and exact *p* values are provided as a Source Data file.

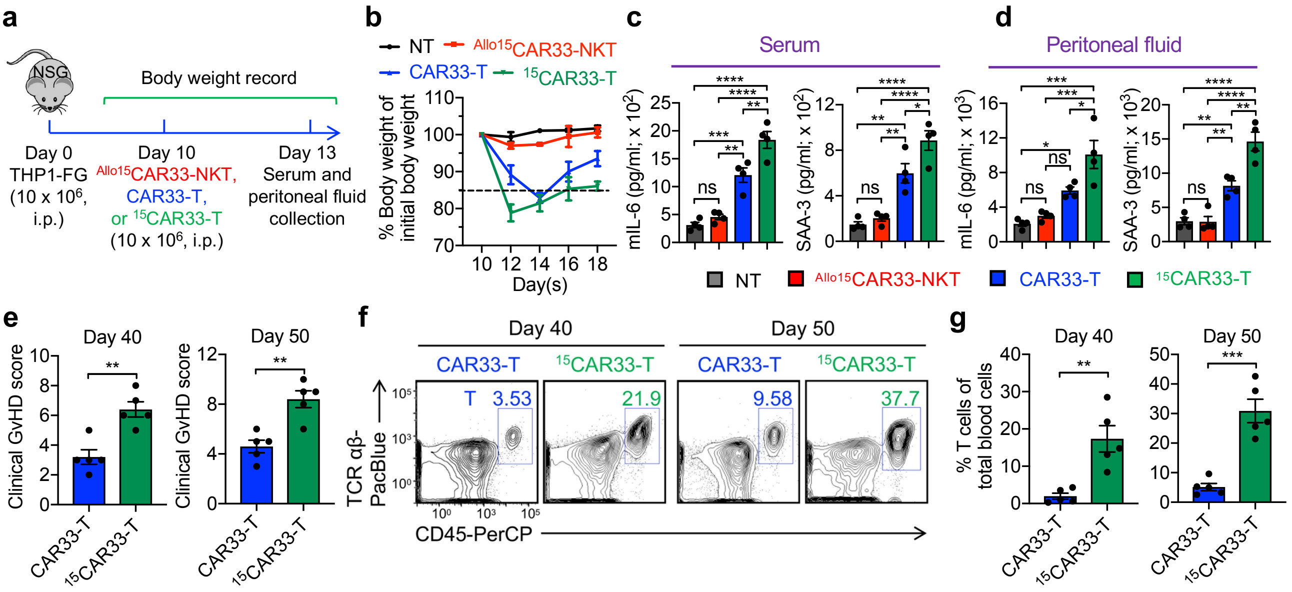

Supplementary Fig. 15. Safety study of ^{15}CAR33-T cells, related to Fig. 10.

a-**f**. Studying the CRS response induced by ^{15}CAR33-T cells using a human THP1-FG xenograft NSG mouse model. **a**. Experimental design. **b**. Body weight of experimental mice over time (n = 4). **c** and **d**. ELISA analyses of mouse IL-6 and SAA3 in mouse serum (**c**) or peritoneal fluid (**d**) (n = 4). NT, samples collected from tumor-bearing mice receiving no therapeutic cell treatment.

e-**g**. Studying the GvHD risk of ^{15}CAR33-T cells using a human THP1-FG xenograft NSG mouse model. **e**. Clinical GvHD score of experimental mice at days 40 and 50 (n = 5). A clinical GvHD score was calculated as the sum of individual scores of 6 categories (body weight, activity, posture, skin thickening, diarrhea, and dishevelment; score 0-2 for each category). **f**. FACS detection of CAR-T cell percentage among total mononuclear cells in mouse blood collected at days 40 and 50. **g**. Quantification of **f** (n = 5).

Representative of 2 experiments. Data are presented as the mean ± SEM. ns, not significant; $*p < 0.05$; $**p < 0.01$; $***p < 0.001$; $****p < 0.0001$, by Student's *t* test (**e** and **g**), or by one-way ANOVA (**c** and **d**). Source data and exact *p* values are provided as a Source Data file.

Supplementary Table 1. Information on clinical primary AML and MDS patient samples.

ID	AML #1	AML #2	AML #3	AML #4	AML #5	AML #6	AML #7	MDS #1
**Diagnosis**	AML progressed from MDS	AML with monocytic differentiation	AML	AML	AML with myelodysplasia	AML with myelodysplasia	AML progressed from MDS	MDS
**Risk Category**	Adverse	Adverse	Adverse	Adverse	Adverse	Adverse	Adverse	Very high risk
**Disease Status**	Untreated	Untreated	Untreated	Untreated	Relapsed/ refractory	Untreated	Untreated	Treated with 2 cycles of azacitidine
**Specimen Source**	Bone marrow (BM)	BM	BM	BM	BM	BM	BM	BM
**Percent Blasts**	30%	65%	50-55%	55%	10-12%	70-80%	36%	No excess blasts
**Karyotype**	44,X,-X,der(3;5)(q10;p10),der(4)t(4;12)(q31.1;q12),add(7)(q11.2),i(11)(q10),-12,der(13)t(1;13)(p22;q34),+mar[20]	43,X,-Y,-2,-3,add(4)(q25),del(4)(p16p12),-5,add(7)(q22),add(11)(q23),-12,add(21)(q22),+2mar[5]/43,idem,del(15)(q11.2q15)[15]	46,XY,+15,der(15;17)(q10;q10)[4]/47,sl,+11[4]/48,sdl1,+13[4]/47,XY,+8[4]/46,XY[4]	46,XY,t(6;9)(p22;q34)[8]/51,idem,+8,+8,+13,+14,+22[12]	46,XY,i(17)(q10)[4]/47,idem,+13[16]	46,XX,inv(3)(q21q26.2)[1]/45,idem,-7[19]	46,XY[20]	45,XX,-7[2]/46,XX[18]
**FISH (Percent of Nuclei)**	+1p (74.5%), +1 (12%), -5q (79.5%), monosomy 5 (5.5%), +5p (7.5%), -7q (76%), +7 (7%), +11q (MLL) (63.5%), -11q (MLL) (6%), -17 (9.5%)	-5q (96.5%), -7q (100%), -21q (RUNX1) (97.5%), +11q (MLL) (96%)	+8q (14.5%), +11q (15%), +15q (29%), -17p (TP53) (24%)	+8q22 (30.5%), +22q11.2 (36.5%)	+13 (64.5%), -17p (TP53) (91.5%), +17q (68%)	EVI1 (MECOM) rearrangement (85.5%), -7 (87%)	Negative	-7 (31%)
**FLT3-ITD**	Negative	Negative	Negative	Positive, VAF 15% (allelic ratio 0.18)	Negative	Negative	Negative	N/A
**Mutations**	TP53	TP53	DNMT3A, GATA2, ISH2, SRSF2, TP53	CEBPA, NRAS	ETV6, SETBP1, SRSF2, ZRSR2	ETV6, NRAS, SF3B1	ASXL1, IDH1, RUNX1, RUNX1, SRSF2	ASXL1, BCOR, DNMT3A, SETBP1

Supplementary Table 2. Cancer stem cell (CSC) and natural killer receptor (NKR) ligand gene signatures

Gene signatures	Gene lists
CSC	*ALCAM*, *ALDH1A1*, *ANPEP*, *BMI1*, *CD151*, *CD24*, *CD44*, *CD47*, *CEACAM6*, *EGFR*, *ENG*, *EPCAM*, *FUT4*, *KIT*, *KLF4*, *LGR5, MSI1*, *MYC*, *MYD88, NANOG*, *POU5F1*, *PROM1*, *SALL4*, *SDC1*, *SLC39A4*, *SOX2*, *THY1*
NKR	*BAG6*, *CD2*, *CD48*, *CFP*, *CLEC2B*, *HLA-C*, *HLA-E*, *HLA-G*, *ICAM1*, *ICAM2*, *ICAM3*, *KMT2E*, *MICA*, *MICB*, *NCR3LG1*, *NECTIN1*, *NECTIN2*, *NECTIN3*, *NID1*, *PCNA*, *PVR*, *RAET1E*, *RAET1G*, *RAET1L, SLAMF6*, *SLAMF7*, *ULBP1*, *ULBP2*, *ULBP3*
