# Supplementary material for: Allogeneic CD33-directed CAR-NKT cells for the treatment of bone marrow-resident myeloid malignancies
Source: Nat Commun. 2025 Feb 1;16:1248. doi: 10.1038/s41467-025-56270-6 (PMC11787387; doi:10.1038/s41467-025-56270-6)
Supplement: Supplementary file 2 — Reporting Summary [file 41467_2025_56270_MOESM2_ESM.pdf]

nature portfolio

Corresponding author(s): Lili Yang

Last updated by author(s): Jan 1, 2025

# Reporting Summary

Nature Portfolio wishes to improve the reproducibility of the work that we publish. This form provides structure for consistency and transparency in reporting. For further information on Nature Portfolio policies, see our Editorial Policies and the Editorial Policy Checklist.

## Statistics

For all statistical analyses, confirm that the following items are present in the figure legend, table legend, main text, or Methods section.

| n/a | Confirmed | |
|---|---|---|
| ☐ | ☒ | The exact sample size (*n*) for each experimental group/condition, given as a discrete number and unit of measurement |
| ☐ | ☒ | A statement on whether measurements were taken from distinct samples or whether the same sample was measured repeatedly |
| ☐ | ☒ | The statistical test(s) used AND whether they are one- or two-sided<br>*Only common tests should be described solely by name; describe more complex techniques in the Methods section.* |
| ☐ | ☒ | A description of all covariates tested |
| ☐ | ☒ | A description of any assumptions or corrections, such as tests of normality and adjustment for multiple comparisons |
| ☐ | ☒ | A full description of the statistical parameters including central tendency (e.g. means) or other basic estimates (e.g. regression coefficient) AND variation (e.g. standard deviation) or associated estimates of uncertainty (e.g. confidence intervals) |
| ☐ | ☒ | For null hypothesis testing, the test statistic (e.g. *F*, *t*, *r*) with confidence intervals, effect sizes, degrees of freedom and *P* value noted<br>*Give P values as exact values whenever suitable.* |
| ☒ | ☐ | For Bayesian analysis, information on the choice of priors and Markov chain Monte Carlo settings |
| ☒ | ☐ | For hierarchical and complex designs, identification of the appropriate level for tests and full reporting of outcomes |
| ☒ | ☐ | Estimates of effect sizes (e.g. Cohen's *d*, Pearson's *r*), indicating how they were calculated |

*Our web collection on statistics for biologists contains articles on many of the points above.*

## Software and code

Policy information about availability of computer code

| | |
|---|---|
| Data collection | MACSQuant Analyzer 10 Flow Cytometer, Illumina NovaSeq 6000 sequencer, Infinite M1000 microplate reader (Tecan), Spectral Advanced Molecular Imaging (AMI) HTX imaging system (Spectral instrument Imaging), ChemiDoc™ Imaging Systems (BIO-RAD) |
| Data analysis | FlowJo software (version 9), AURA imaging software (version 3.2.0), Graphpad Prism (version 8), Image J (version 1.53s), R package Seurat (v.4.0.0) |

For manuscripts utilizing custom algorithms or software that are central to the research but not yet described in published literature, software must be made available to editors and reviewers. We strongly encourage code deposition in a community repository (e.g. GitHub). See the Nature Portfolio guidelines for submitting code & software for further information.

## Data

Policy information about availability of data

All manuscripts must include a data availability statement. This statement should provide the following information, where applicable:

- Accession codes, unique identifiers, or web links for publicly available datasets
- A description of any restrictions on data availability
- For clinical datasets or third party data, please ensure that the statement adheres to our policy

All data associated with this study are present in the paper or Supplemental Information. The genomics data generated during this study are available from the public repository Gene Expression Omnibus Database: GSE253982 (scTCR-seq, related to Fig. 2h), and GSE245375 (scRNAseq, related to Fig. 6

a). Additional information and materials will be made available upon reasonable request.

## Research involving human participants, their data, or biological material

Policy information about studies with human participants or human data. See also policy information about sex, gender (identity/presentation), and sexual orientation and race, ethnicity and racism.

| | |
|---|---|
| Reporting on sex and gender | Sex and gender are not considered in the design of this study. |
| Reporting on race, ethnicity, or other socially relevant groupings | Race, ethnicity, or other socially relevant groupings are not considered in the design of this study. |
| Population characteristics | Primary acute myeloid leukemia (AML) and myelodysplastic syndromes (MDS) patient bone marrow samples were collected at the Ronald Reagan UCLA Medical Center from consented patients through an IRB-approved protocol (IRB#22-000558) and processed. |
| Recruitment | Healthy donor PBMCs were obtained from the UCLA/CFAR Virology Core Laboratory. Primary AML and MDS patient bone marrow samples were collected at the Ronald Reagan UCLA Medical Center from consented patients and processed. |
| Ethics oversight | Healthy donor PBMCs were obtained from the UCLA/CFAR Virology Core Laboratory without identification information under federal and state regulations. Primary AML and MDS patient bone marrow samples were collected at the Ronald Reagan UCLA Medical Center from consented patients through an IRB-approved protocol (IRB#22-000558). |

Note that full information on the approval of the study protocol must also be provided in the manuscript.

# Field-specific reporting

Please select the one below that is the best fit for your research. If you are not sure, read the appropriate sections before making your selection.

[x] Life sciences   [ ] Behavioural & social sciences   [ ] Ecological, evolutionary & environmental sciences

For a reference copy of the document with all sections, see nature.com/documents/nr-reporting-summary-flat.pdf

# Life sciences study design

All studies must disclose on these points even when the disclosure is negative.

| | |
|---|---|
| Sample size | For all studies samples size were defined on the basis of previous studies in the laboratory (PMID: 34841295, 37938576, and 34112755). For in vivo studies, between 3 to 10 mice per group were used dependent on the experimental design and were able to reach a significant level of 0.05. For in vitro studies, at least 3 biological group were used as cell donors with technical replicates. For studies involving primary patient samples, at least 3 patient samples per group were used. |
| Data exclusions | No data were excluded from the analyses. |
| Replication | All experiments were performed independently. Replicates of each individual experiment was stated in its figure legends. The experimental findings were reproduced with similar results. For single cell RNA sequencing, a mixture of 10 experimental mice were combined for each group. |
| Randomization | For in vivo studies, all mice received tumor cells at Day 0 then were randomized to split into different groups prior to therapeutic cells injection. All the in vitro and in vivo experiments each group was selected randomly. |
| Blinding | Experiments were not performed in a blinded fashion. All data were analyzed by software with objective standard. |

# Reporting for specific materials, systems and methods

We require information from authors about some types of materials, experimental systems and methods used in many studies. Here, indicate whether each material, system or method listed is relevant to your study. If you are not sure if a list item applies to your research, read the appropriate section before selecting a response.

## Materials & experimental systems

| n/a | Involved in the study |
| --- | --- |
| ☐ | ☒ Antibodies |
| ☐ | ☒ Eukaryotic cell lines |
| ☒ | ☐ Palaeontology and archaeology |
| ☐ | ☒ Animals and other organisms |
| ☒ | ☐ Clinical data |
| ☒ | ☐ Dual use research of concern |
| ☒ | ☐ Plants |

## Methods

| n/a | Involved in the study |
| --- | --- |
| ☒ | ☐ ChIP-seq |
| ☐ | ☒ Flow cytometry |
| ☒ | ☐ MRI-based neuroimaging |

# Antibodies

Antibodies used

Fluorochrome-conjugated antibodies specific for human CD45 (Clone HI30, PerCP, FITC, or Pacific Blue-conjugated, 1:500, cat. no. 982318, 982316, or 982306), TCR αβ (Clone IP26, Pacific Blue or PE-Cy7-conjugated, 1:25, cat. no. 306716 or 306720), CD3 (Clone HIT3a, Pacific Blue, PE, or PE-Cy7-conjugated, 1:500, cat. no. 300330, 300308, or 300316), CD4 (Clone OKT4, PE-Cy7, PerCP or FITC-conjugated, 1:500, cat. no. 317414, 317432 or 317408), CD8 (Clone SK1, PE, APC-Cy7, or APC-conjugated, 1:300, cat. no. 344706, 344714 or 344722), CD8β (Clone QA20A40, PE-Cy7-conjugated, 1:500, cat. no. 376708), CD14 (Clone HCD14, Pacific Blue-conjugated, 1:100, cat. no. 367122), CD15 (Clone W6D3, APC-conjugated, 1:500, cat. no. 323008), CD19 (Clone HIB19, APC-Cy7-conjugated, 1:200, cat. no. 302218), CD20 (Clone 2H7, APC-Cy7-conjugated, 1:200, cat. no. 302314), CD33 (Clone WM53, APC or PE-Cy7-conjugated, 1:100, cat. no. 983902 or 983908), CD34 (Clone 581, PerCP- conjugated, 1:500, cat. no. 343520), CD38 (Clone HIT2, Pacific Blue-conjugated, 1:200, cat. no. 980316), CD56 (Clone HCD56, FITC or PerCP-conjugated, 1:10, cat. no. 318304 or 318342), CD69 (Clone FN50, PE-Cy7 or PerCP-conjugated, 1:50, cat. no. 310912 or 310928), CD1d (Clone 51.1, PE-Cy7 or APC-conjugated, 1:50, cat. no. 350310 or 350308), CD112 (Clone TX31, PE-conjugated, 1:250, cat. no. 337410), CD155 (Clone SKII.4, PE-Cy7-conjugated, 1:250, cat. no. 337614), CD11b (Clone ICRF44, FITC-conjugated, 1:500, cat. no. 982614), MICA/MICB (Clone 6D4, PE or APC-conjugated, 1:25, cat. no. 320906 or 320908), 41BBL (Clone 5F4, PE-conjugated, 1:500, cat. no. 311504), CD83 (Clone HB15e, APC-Cy7-conjugated, 1:500, cat. no. 305330), CD86 (Clone IT2.2, APC-conjugated, 1:500, cat. no. 305412), PD-1 (Clone A17188A, PE or FITC-conjugated, 1:25, cat. no. 379210 or 379206), TIM-3 (Clone A18087E, APC-conjugated, 1:25, cat. no. 364804), LAG-3 (Clone 7H2C65, PE-Cy7-conjugated, 1:25, cat. no. 369208), NKG2D (Clone 1D11, PE-Cy7-conjugated, 1:50, cat. no. 320812), DNAM-1 (Clone 11A8, APC-conjugated, 1:50, cat. no. 338312), NKp30 (Clone P30-15, APC-conjugated, 1:50, cat. no. 325210), NKp46 (Clone 9E2, FITC-conjugated, 1:50, cat. no. 137606), CXCR4 (Clone 12G5, PE-Cy7-conjugated, 1:50, cat.no. 306514), CCR5 (Clone J418F1, APC-Cy7-conjugated, 1:50, cat. no. 359110), SOX2 (Clone 14A6A34, Pacific Blue-conjugated, 1:200, cat. no. 656112), OCT3/4 (Clone 3A2A20, PE-conjugated, 1:200, cat. no. 653704), IFN-γ (Clone B27, PE-Cy7-conjugated, 1:50, cat. no. 506518), Granzyme B (Clone QA16A02, APC-conjugated, 1:2000 or 1:5000, cat. no. 372204), Perforin (Clone dG9, PE-Cy7-conjugated, 1:50 or 1:100, cat. no. 308126), TNF-α (Clone MAb11, APC-conjugated, 1:4000, cat. no. 502912), IL-2 (Clone MQ1-17H12, APC-Cy7-conjugated, 1:50, cat. no. 500342), β2-microglobulin (B2M) (Clone 2M2, FITC or APC-conjugated, 1:2000 or 1:5000, cat. no. 316304 or 316311), HLA-DR (Clone L243, APC-Cy7-conjugated, 1:200 or 1:500, cat. no. 307618), and HLA-DR, DP, DQ (Clone Tü39, FITC-conjugated, 1:200 or 1:500, cat. no. 361706) were purchased from BioLegend. Fluorochrome-conjugated antibodies specific for human iNKT TCR Vα24-Jβ18 (Clone 6B11, PE-conjugated, 1:20, cat. no. 552825) were purchased from BD Biosciences. Fluorochrome-conjugated antibody specific for human iNKT TCR Vβ11 (Clone C21, APC-conjugated, 1:50, cat. no. IM2290) was purchased from Beckman-Coulter. Fluorochrome-conjugated antibodies specific for human ULBP-1 (Clone 170818, PE-conjugated or unconjugated, 1:25, cat. no. FAB1380P or MAB1380) and ULBP-2,5,6 (Clone 165903, APC-conjugated, 1:25, cat. no. FAB1298A) were purchased from R&D Systems. A goat anti-mouse IgG F (ab')2 secondary antibody was purchased from ThermoFisher (cat. no. A-11001). Fixable Viability Dye eFluor506 (e506, 1:500, cat. no. 65-0866-14) was purchased from Affymetrix eBioscience; mouse Fc Block (anti-mouse CD16/32, cat. no. 553141) was purchased from BD Biosciences; and human Fc Receptor Blocking Solution (TrueStain FcX) was purchased from BioLegend (cat. no. 422302). In our study, note the use of antibodies with identical clones but differing conjugated fluorochromes, with one typical antibody listed herein.

Mouse IL-6 was quantified with paired purified anti-mouse IL-6 antibody (Clone MP5-20F3, cat. no. 504501) and biotin anti-mouse IL-6 antibody (Clone MP5-32C11, cat. no. 504601) (BioLegend).

Validation

All the antibodies used are from commercial sources and have been validated by the vendors. Validation data are available on the manufacturer's website. Each antibody purchased for this study has relevant citation and profile.

# Eukaryotic cell lines

Policy information about cell lines and Sex and Gender in Research

Cell line source(s)

Human acute myeloid leukemia cell line THP1 (cat. no. TIB-202), KG1 (cat. no. CCL-246), and HL60 (cat. no. CCL-240) were purchased from the American Type Culture Collection (ATCC). All cell lines used in this study were tested mycoplasma free.

Authentication

Human acute myeloid leukemia cell line THP1, KG1, and HL60 cells were authenticated by ATCC.
All the FG engineered cells and CD33 knockout cells were not authenticated.

Mycoplasma contamination

All cell lines were tested negative for mycoplasma contamination.

Commonly misidentified lines
(See ICLAC register)

No misidentified lines were used.

## Animals and other research organisms

Policy information about studies involving animals; ARRIVE guidelines recommended for reporting animal research, and Sex and Gender in Research

| | |
|---|---|
| Laboratory animals | NOD.Cg-PrkdcSCIDIl2rgtm1Wjl/SzJ (NOD/SCID/IL-2Rγ-/-, NSG) mice were maintained in the animal facilities of the University of California, Los Angeles (UCLA) under the following housing conditions: temperature ranging from 68°F to 79°F, humidity maintained at 30% to 70%, a light cycle of On at 6:00 am and Off at 6:00 pm, and room pressure set to negative. 6-10 weeks old female mice were used for all experiments unless otherwise indicated. |
| Wild animals | No wild animals were used. |
| Reporting on sex | 6-10 weeks old male or female mice were used for all experiments unless otherwise indicated. |
| Field-collected samples | This study did not involve field-collected samples. |
| Ethics oversight | All animals were maintained at the UCLA animal facilities and all animal experiments were approved by the Institutional Animal Care and Use Committee of UCLA. |

Note that full information on the approval of the study protocol must also be provided in the manuscript.

## Plants

| | |
|---|---|
| Seed stocks | NA |
| Novel plant genotypes | NA |
| Authentication | NA |

## Flow Cytometry

### Plots

Confirm that:

- [x] The axis labels state the marker and fluorochrome used (e.g. CD4-FITC).
- [x] The axis scales are clearly visible. Include numbers along axes only for bottom left plot of group (a 'group' is an analysis of identical markers).
- [x] All plots are contour plots with outliers or pseudocolor plots.
- [x] A numerical value for number of cells or percentage (with statistics) is provided.

### Methodology

| | |
|---|---|
| Sample preparation | Purified cord blood derived human CD34+ cells were purchased from the HemaCare. Healthy donor PBMCs were obtained from the UCLA/CFAR Virology Core Laboratory without identification information under federal and state regulations. Primary AML and MDS patient bone marrow samples were collected at the Ronald Reagan UCLA Medical Center from consented patients through an IRB-approved protocol (IRB#22-000558) and processed. Allo15CAR33-NKT cells were generated by differentiating gene-engineered cord blood CD34+ HSPCs in a 5-stage Ex Vivo HSC-Derived CAR-NKT Cell Culture. Healthy donor PBMCs were used to generate the PBMC-derived conventional αβ T (PBMC-Tc). In mouse study, cells were collected from mouse organs (e.g., bone marrow, spleen, liver, lung, and blood). |
| Instrument | MACSQuant Analyzer 10 flow cytometer (Miltenyi Biotech) |
| Software | FlowJo software version 9 (BD Biosciences) |
| Cell population abundance | For cell sorting, highest purity method was used in FACSAria II Flow Cytometer and over 98% post-sorting purity was verified through flow cytometry analysis. |

| | |
|---|---|
| Gating strategy | SSC-A/FSC-A, SSC-W/SSC-H and FSC-W/FSC-H were used to identify singlets. Fixable Viability Dye eFluor 506 (Biolegend) was used to exclude dead cells in flow analysis. DAPI (Thermo Fisher Scientific) was included to exclude dead cells in FACS sorting. Gating strategy for each cell subtype in each experiment was described in the article and provided as panels in supplementary figures. |

☒ Tick this box to confirm that a figure exemplifying the gating strategy is provided in the Supplementary Information.
